# Incidence of arrhythmias in COVID-19 patients with double mutant strain of SARS-CoV-2 virus: A tertiary care experience

**DOI:** 10.21542/gcsp.2022.16

**Published:** 2022-12-30

**Authors:** Amit Varshney, Navneet Agarwal

**Affiliations:** Department of Medicine, United Institute of Medical Sciences, Prayagraj, U.P., India

## Abstract

**Background:** Our understanding of arrhythmias is minimal with SARS-CoV-2 virus and with the emergence of its double mutant, virtually nonexistent. Patients with the double mutant (B.1.617) SARS-CoV infection had more cardiac manifestations, including arrhythmias and sudden death, than with the traditional variant.

**Objective:** To determine the incidence of arrhythmias in COVID-19 patients with double mutant strain of SARS-CoV-2 virus (B.1.617).

**Materials and methods:** We describe a prospective observational study conducted in the Department of Medicine, United Institute of Medical Sciences, Prayagraj, Uttar Pradesh on patients admitted to the hospital during the period March 2021 to May 2021. Different type of arrhythmias were studied in the admitted patients.

**Results:** Sinus bradycardia is the most common arrhythmia, followed by atrial fibrillation. Malignant arrhythmias, such as ventricular tachycardia/ventricular fibrillation and Torsades de pointes due to QT prolongation, were present in small number of patients with high mortality outcomes. Sinus tachycardia and high-grade AV blocks were also present in some of the patients.

**Conclusions:** Current literature lacks studies on arrhythmias secondary to COVID-19 (double mutant) strain and its possible mechanisms. This makes it difficult to distinguish between arrhythmias secondary to COVID-19 (double mutant) infection due to hypoxemia, dyselectrolytemia, SIRS, comorbidities, and medications or direct viral effects on the cardiomyocytes.

## Introduction

India is in the midst of a devastating second wave of COVID-19. For the past several weeks, cases and deaths have reached a new height. The country is recording more than 400,000 cases per day^1^.

The situation in India sounds quite similar to what has already happened in Brazil, and South Africa but then, over the course of time, as people’s immunity waned, more contagious variants surfaced and sparked another surge. The latest of these variants been labeled as B.1.617 (double mutant). This subtype has two key mutations that have cropped up in two other infamous strains.

There are well-documented cardiac complications of COVID-19 in patients with and without prior CVD. Myocardial injury is very common, especially in critically ill COVID-19-infected patients, through different mechanisms mainly due to direct damage of cardiomyocytes and SIRS (systemic inflammatory response syndrome). Cardiac complications include myocarditis, heart failure, and acute coronary syndrome resulting from coronary artery thrombosis or SARS-CoV-2-related plaque disruption^2^.

Our understanding of arrhythmias is minimal with SARS-CoV-2 virus and with the emergence of its double mutant, virtually nonexistent.

Evidence has shown that arrhythmias are also one of the major cardiac complications. Liu et al. reported that about 7% of patients reported palpitations as a presenting symptom. In a recent report from Wuhan, China, 16.7% of hospitalized and 44.4% of ICU patients with COVID-19 had cardiac arrhythmias^3^.

The probable mechanisms for arrhythmogenicity in COVID-19 include altered intercellular coupling, interstitial edema, and cardiac fibrosis that leads to abnormal conduction in addition to abnormal Ca^2+^ handling and downregulation of K^+^ channels - resulting in repolarization abnormalities and action potential conduction abnormalities^4^.

Patients with the double mutant SARS-CoV infection had more cardiac manifestations, including arrhythmias and sudden death, than its traditional variant.

## Material and Methods

We conducted this study in Department of Medicine, United Institute of Medical Sciences, Prayagraj, Uttar Pradesh on 43 patients (32 in ICU and 11 in General Medicine ward) admitted to the hospital during the period March 2021 to May 2021.

The inclusion criteria were:

1. Age >18 years
2. COVID disease due to variant B.1.617 (double mutant),

The exclusion criteria were:

1. Patients who do not give consent
2. Age <18 years
3. COVID disease due to traditional variant
4. Patients suffering from end stage renal and hepatic diseases
5. Pregnant females
6. Patients who had prior history of arrhythmias
7. Patients who had thyroid disorders

Double mutant strain infection was confirmed by sending samples to NIV (National Institute of Virology), Pune, India.

## Results

**Figure 1.**
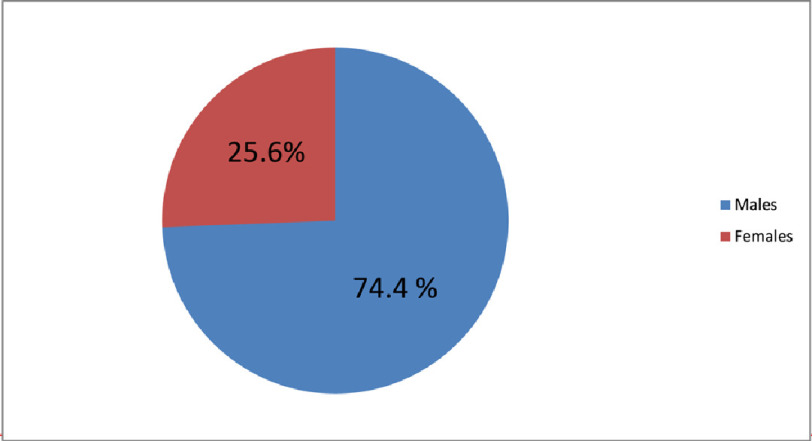
Sex distribution in cases of arrhythmias in patients infected with COVID-19 double mutant strain.

In our study the mean age of participants was 40.2 ± 12.2 years, of which 32 (74.4%) were males and 11(25.6%) were females (Figure 1).

Sinus bradycardia is the most common arrhythmia which was found in 44% patients (*n* = 19: 16 males, 3 females), followed by atrial fibrillation which was present in 23% patients (*n* = 10: 7 males, 3 females) (Table 1).

**Table 1:** Distribution of patients with arrythmias according to their sex.

**Arrhythmias**	TOTAL n (%)	Males n (%)	Females n (%)
Sinus bradycardia	19 (44%)	16 (37%)	3 (7%)
Atrial fibrillation	10 (23%)	7 (16%)	3 (7%)
Sinus tachycardia	6 (14%)	4 (9%)	2 (5%)
VT/VF	4 (9%)	2 (5%)	2 (5%)
Torsades de pointes	2 (5%)	1 (2%)	1 (2%)
High Grade AV Block	2 (5%)	2 (5%)	–

**Figure 2.**
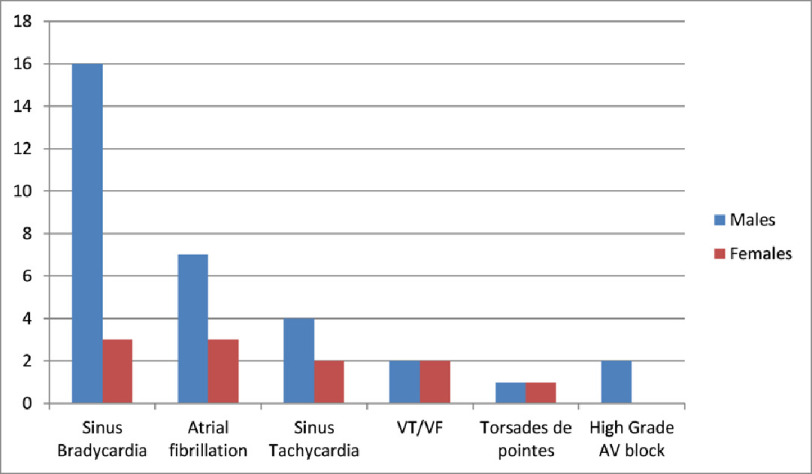
Distribution of arrhythmias in patients according to their gender.

Malignant arrhythmias such as ventricular tachycardia/ventricular fibrillation were present in 9% patients (*n* = 4: 2 males and 2 females) (Figure 2).

Torsade de pointes due to QT prolongation was present in 5% patients (*n* = 2: 1 male and 1 female).

Sinus tachycardia was present in about 14% patients (*n* = 6: 4 males and 2 females).

**Figure 3.**
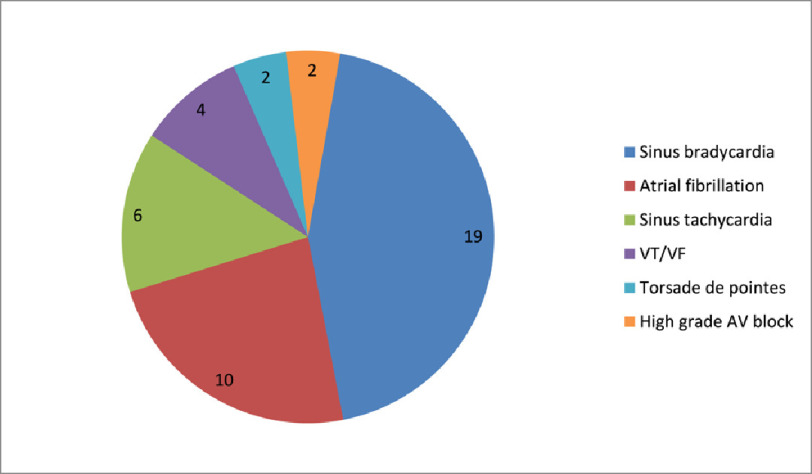
Distribution of patients with specific arrythmias.

High-grade AV blocks were present in 5% of patients (*n* = 2: both males) (Figure 3).

## Discussion

Our understanding of arrhythmic complications in COVID-19 (double mutant strain) is still evolving. We have an increasing number of cases of arrhythmias at our center, but there is very little literature on arrhythmias in COVID-19 patients, especially in double mutant strain.

Wang et al. reported that among 138 patients who were hospitalized with COVID-19, arrhythmias were reported in 17% of the patients and more commonly in 44% of the patients admitted to an intensive care unit^5^. In a group of 393 patients with COVID-19 patients in New York, rates of atrial arrhythmias were higher among patients requiring mechanical ventilation (17.7% in mechanically ventilated patients compared with 1.9% in non-invasive ventilation groups)^6^.

The most common arrhythmia we found in relation to COVID-19 double mutant strain was sinus bradycardia (19 patients), which was found mainly in our wards. In a report by Kir et al., bradycardias were seen in a patient with COVID-19 infection with normal echocardiography and normal cardiac biomarkers^7^.

Atrial fibrillation was the second most common cardiac arrhythmia observed in patients with COVID-19 infection (double mutant strain), found in our 23% of patients. Most of these patients were in intensive care units, which is the same as found by Gopinathannair et al. in their survey, and also by Seecheran et al. ^8^.

Patients who developed atrial fibrillation were typically older in age (mean age 59 ± 12.2 years). The mechanisms that might cause atrial fibrillation in these patients are elusive, mainly due to systemic infection, direct viral cardiomyocyte injury, hypoxemia, susceptibility of the population due to advanced age and their comorbidities, and, finally, sympathetic nervous system overactivity^9^.

Additional atrial and ventricular arrhythmias have been witnessed in COVID-19 double mutant strain patients, without any prior history of arrhythmia or heart disease.

Malignant ventricular arrhythmias, which consist of ventricular tachycardia/ventricular fibrillation, were found in about 9% of patients who were mostly mechanically ventilated and had very high cardiac markers. In COVID-19 double mutant strain, malignant ventricular arrhythmias could be secondary to the side effects of medication, hypoxia, pulmonary disease, activated protein kinase C, direct oxidized Ca2+/calmodulin-dependent protein kinase II activity and myocarditis^10^.

Torsade de pointes due to QT-prolongation was found in 5% of patients who were advanced in age, on mechanical ventilation, and had a history of intake of medications causing QT- prolongation, such as azithromycin and hydroxychloroquine, which was similar to previous reports^11–13^.

Cardiac arrhythmias had also been associated with disease severity. It had been seen that patients with elevated troponin T levels were at higher risk of severe disease, ICU admission, and death. Furthermore, new-onset arrhythmia, elevated biomarkers including CPK-MB, lactate dehydrogenase (LDH), inflammatory biomarkers including C-reactive protein (CRP), and IL-6 levels were all associated with severe disease^14,15^.

High-grade AV blocks were present in 5% of our patients, which may be due to SARS-CoV-2 tropism for cardiac myocytes and the inflammatory response of the host. Virus replicating within myocytes provokes an adaptive immune response characterized by an influx of natural killer cells and T cells^16^. These cells are ultimately responsible for viral clearance, but in the process cause cytokine-mediated cellular damage and edema that result in interruption of the conduction pathways^17,18^.

Further studies should be done to determine whether patients with viral myocarditis secondary to COVID-19 double mutant strain infection are at an increased risk of lethal arrhythmias when given arrhythmiogenic medications, as compared to patients receiving the same medications without evidence of viral myocarditis.

## Conclusion

Many viral infections are known to cause arrhythmias due to viral myocarditis and existing anecdotal evidence suggests this can also occur with double mutant strain of SARS-CoV-2 virus (B.1.617) patients as well.

Current literature lacks studies on arrhythmias secondary to COVID-19 (double mutant) strain and its possible mechanisms. This makes it difficult to distinguish between arrhythmias secondary to COVID-19 (double mutant) infection due to hypoxemia, dyselectrolytemia, SIRS, co-morbidities, and medications or direct viral effects on the cardiomyocytes. In order to find the exact mechanisms and its long-term consequences, further research is required.

## Conflict of Interest

The authors declare that they have no conflict of interest.

References1.[Internet]
https://www.worldometers.info/coronavirus/country/india/
20 May 20212.
Babapoor-FarrokhranS
GillD
WalkerJ
RasekhiRT
BozorgniaB
AmanullahA
2020Myocardial injury and COVID-19: possible mechanismsLife Sci253117723doi: 10.1016/j.lfs.2020.11772332360126PMC71945333.
LiuK
FangY-Y
DengY
LiuW
WangM-F
MaJ-P
XiaoW
WangYN
ZhongMH
LiCH
LiGC
LiuHG
2020Clinical characteristics of novel coronavirus cases in tertiary hospitals in Hubei ProvinceChinese Med J133910251031doi: 10.1097/CM9.0000000000000744PMC7147277320448144.
TseG
YeoJM
ChanYW
LaiETHL
YanBP
2016What is the arrhythmic substrate in viral myocarditis? Insights from clinical and animal studiesFront Physiol7308doi: 10.3389/fphys.2016.0030827493633PMC49548485.
WangD
HuB
HuC
ZhuF
LiuX
ZhangJ
WangB
XiangH
ChengZ
XiongY
ZhaoY
LiY
WangX
PengZ
2020Clinical characteristics of 138 hospitalized patients with 2019 novel coronavirus-infected pneumonia in WuhanChina JAMA3231061doi: 10.1001/jama.2020.158532031570PMC70428816.
GoyalP
ChoiJJ
PinheiroLC
SchenckEJ
ChenR
JabriA
SatlinMJ
Campion JrTR
NahidM
RingelJB
HoffmanKL
AlshakMN
LiHA
WehmeyerGT
RajanM
ReshetnyakE
HupertN
HornEM
 MartinezFJ
GulickRM
SaffordMM
2020Clinical characteristics of Covid-19 in New York CityN Engl J Med38223722374doi: 10.1056/NEJMc201041932302078PMC71820187.
KirD
MohanC
SancassaniR
2020Heart break: an unusual cardiac manifestation of coronavirus disease 2019 (COVID-19)JACC Case Rep212521255doi: 10.1016/j.jaccas.2020.04.02632368756PMC71964138.
GopinathannairR
MerchantFM
LakkireddyDR
EtheridgeSP
FeigofskyS
HanJK

2020COVID-19 and cardiac arrhythmias: a global perspective on arrhythmia characteristics and management strategiesJ Interv Card Electrophysioldoi: 10.1007/s10840-020-00789-9PMC7268965324948969.
RussoV
RagoA
CarboneA
BottinoR
AmmendolaE
Della CioppaN

2020Atrial fibrillation in COVID-19: from epidemiological association to pharmacological implicationsJ Cardiovas Pharmacoldoi: 10.1097/FJC.00000000000008543245307410.
SattarY
UllahW
RaufH
UlH
VirkH
YadavS
ChowdhuryM

2020COVID-19 cardiovascular epidemiology, cellular pathogenesis, clinical manifestations and managementIJC Heart and Vasculature, vol. 29Elsevier Ireland Ltdp. 100589doi: 10.1016/j.ijcha.2020.100589PMC73597943272483111.
ChorinE
DaiM
ShulmanE
WadhwaniL
Bar-CohenR
BarbhaiyaC
AizerA
HolmesD
BernsteinS
SpinelliM
ParkDS
ChinitzLA
JankelsonL
2020The QT interval in patients with COVID-19 treated with hydroxychloroquine and azithromycinNat Med26808809doi: 10.1038/s41591-020-0888-23248821712.
SalehM
GabrielsJ
ChangD
KimBS
MansoorA
MahmoodE

2020The effect of chloroquine, hydroxychloroquine and azithromycin on the corrected QT interval in patients with SARS-CoV-2 infectionCircArrhythmElectrophysiol13e008662doi: 10.1161/CIRCEP.120.008662PMC72990953234774313.
MercuroNJ
YenCF
ShimDJ
MaherTR
McCoyCM
ZimetbaumPJ

2020Risk of QT interval prolongation associated with use of hydroxychloroquine with or without concomitant azithromycin among hospitalized patients testing positive for coronavirus disease 2019 (COVID-19)JAMA Cardioldoi: 10.1001/jamacardio.2020.1834PMC71956923293625214.
AzevedoRB
BotelhoBG
de HollandaJVG
FerreiraLVL
Junqueira de AndradeLZ
OeiSSML

2020Covid-19 and the cardiovascular system: a comprehensive reviewJ Hum Hypertens18doi: 10.1038/s41371-020-0387-4PMC73847293271944715.
LiX
PanX
LiY
AnN
XingY
YangF

2020Cardiac injury associated with severe disease or ICU admission and death in hospitalized patients with COVID-19: a meta-analysis and systematic reviewCrit Care241468doi: 10.1186/s13054-020-03183-z32723362PMC738617016.
KirmserR
UmbachR
RowettD
RossA
1977Complete heart block due to acute nonspecific carditisChest715682684doi: 10.1378/chest.71.5.68285235517.
BatraAS
EpsteinD
SilkaMJ
2003The clinical course of acquired complete heart block in children with acute myocarditisPediatrCardiol24549549710.1007/s00246-002-0402-21462732318.
FeldmanAM
McNamaraD
2000MyocarditisN Engl J Med34319138813981107010510.1056/NEJM200011093431908
